# Spatial relationships between above-ground biomass and bird species biodiversity in Palawan, Philippines

**DOI:** 10.1371/journal.pone.0186742

**Published:** 2017-12-04

**Authors:** Minerva Singh, Daniel A. Friess, Bruno Vilela, Jose Don T. De Alban, Angelica Kristina V. Monzon, Rizza Karen A. Veridiano, Roven D. Tumaneng

**Affiliations:** 1 Department of Plant Sciences, University of Cambridge, Cambridge, United Kingdom; 2 Department of Geography, National University of Singapore, 1Arts Link, Singapore, Singapore; 3 Department of Biology, Washington University in Saint Louis, St. Louis, Missouri, United States of America; 4 Fauna & Flora International, Philippines Programme, Tagaytay City, Cavite, Philippines; 5 Department of Biological Sciences, National University of Singapore, Singapore, Singapore; 6 Department of Geography, University of Cambridge, Cambridge, United Kingdom; 7 Johann Heinrich von Thünen Institute for International Forestry and Forest Economics, Hamburg, Germany; 8 Emerging Technology Development Division, Philippine Council for Industry, Energy, and Emerging Technology Research and Development, Department of Science and Technology, Taguig City, Philippines; University of Brasilia, BRAZIL

## Abstract

This study maps distribution and spatial congruence between Above-Ground Biomass (AGB) and species richness of IUCN listed conservation-dependent and endemic avian fauna in Palawan, Philippines. Grey Level Co-Occurrence Texture Matrices (GLCMs) extracted from Landsat and ALOS-PALSAR were used in conjunction with local field data to model and map local-scale field AGB using the Random Forest algorithm (r = 0.92 and RMSE = 31.33 Mg·ha^-1^). A support vector regression (SVR) model was used to identify the factors influencing variation in avian species richness at a 1km scale. AGB is one of the most important determinants of avian species richness for the study area. Topographic factors and anthropogenic factors such as distance from the roads were also found to strongly influence avian species richness. Hotspots of high AGB and high species richness concentration were mapped using hotspot analysis and the overlaps between areas of high AGB and avian species richness was calculated. Results show that the overlaps between areas of high AGB with high IUCN red listed avian species richness and endemic avian species richness were fairly limited at 13% and 8% at the 1-km scale. The overlap between 1) low AGB and low IUCN richness, and 2) low AGB and low endemic avian species richness was higher at 36% and 12% respectively. The enhanced capacity to spatially map the correlation between AGB and avian species richness distribution will further assist the conservation and protection of forest areas and threatened avian species.

## Introduction

Many tropical forested ecosystems continue to experience rapid decline, which in turn is associated with loss of ecosystem service provisions and biodiversity [1,2,3]. Payments for Ecosystem Services (PES) mechanisms such as Reduced Emissions from Degradation and Deforestation (REDD+) have gained traction as a means of incentivizing tropical forest conservation in order to protect forest carbon stocks or above ground biomass (AGB) that would otherwise be lost to deforestation. There has also been a strong interest in using REDD+ to provide biodiversity co-benefits (such as the conservation of endangered species) [4,5].

While PES may be able to provide biodiversity conservation co-benefits, overlaps between AGB and faunal species richness vary across tropical forest ecosystems and are variable at different scales [6]. A global study discovered significant overlaps between AGB and avian species richness, especially when threatened-only species were considered [7]. However, overlaps between AGB and biodiversity may be weak or non-existent at smaller scales in different tropical forest ecosystems. An examination of congruence between AGB and biodiversity was conducted across the different islands of Indonesia [8], where variable but weak overlaps were discovered between AGB and biodiversity. These findings were further validated by a study conducted across 14 different tropical sites across South America, Africa and Indonesia, where limited overlap was observed between mammal and avian species richness and AGB [9]. Conversely, previous research has shown that AGB plays an important role in explaining avian species richness across a degradation gradient in the Amazon [10]. On the basis of these findings, it is imperative to examine the strength of associations that exist between AGB and species richness regionally and at local scales in different regions [11]. However, our lack of knowledge regarding the overlaps and co-existence of AGB stocks, biodiversity rich areas, and suitable habitats in this landscape is a major hindrance in devising PES mechanisms which could facilitate both carbon and biodiversity conservation.

Remote sensing plays an important role in mapping AGB and carbon at landscape-scale in the tropics [12,13,14]. Data from multiple sensors has been used in conjunction with field data from different tropical countries to produce a global AGB map at 500-m resolution [15]. Subsequent examination of this global scale map revealed that it can capture biome or country scale spatial variations in AGB moderately well [16]. The introduction of global scale AGB maps does not eliminate the need for higher resolution local or national scale AGB maps; on the contrary, local AGB maps and field data can inform and improve the efficacy of global scale medium resolution AGB estimates [17].

Both optical and radar based systems such as Landsat and ALOS-PALSAR offer the potential for AGB monitoring at multiple scales [18]. However, optical and radar data undergo saturation at higher AGB values. Image texture derived variables, particularly those extracted using grey level co-occurrence matrix or GLCM have been extensively used for AGB mapping and have the potential to overcome AGB saturation, especially in degraded and disturbed forests [19,20]. Further, GLCM texture measures derived from Synthetic Aperture Radar (SAR) data have also been used to accurately model AGB in Malaysia, Thailand and Brazil [21]. Inclusion of texture measures derived from radar has also helped produce robust AGB estimates in a mixed forest ecosystem in Sumatra [22]. Hence, it is expected that a combination of optical and radar data will help us map and monitor AGB stocks at landscape/sub-national scale in inaccessible tropical rainforests.

Avian species richness is a widely used indicator of biodiversity value, and it is particularly suitable for mapping because changes in patterns of avian species richness and diversity tie in well with larger landscape-level habitat metrics that can be mapped [23]. Avian species richness is influenced by multiple abiotic and biotic factors that vary considerably across different spatial scales. At smaller scales, avian species richness is influenced by factors such as habitat diversity, whereas at larger scales, energy and bio-climatic variables such as temperature become more important [24]. Even at the resolution of 1 degree (111.32 km), topography and temperature are more important determinants of global scale avian diversity [25]. While habitat characteristics influence species richness at finer scales [26], the role of bioclimatic and topographic variables cannot be eliminated [27]. In addition, species richness is influenced by anthropogenic factors such as distance from roads and forest management regimes [28].

While a combination of habitat conditions, human disturbance, and bioclimatic variables are known to influence avian species richness [29], only a handful of studies have examined the role of AGB in influencing avian species richness at a regional level [10,8,9]. These too produce a conflicting picture of AGB and avian species richness interactions and overlaps. Previous research on AGB and biodiversity distribution in the Philippines suggests there is potential synergy between REDD+ and biodiversity conservation schemes [30]. Identifying the role of AGB in influencing avian species richness and the interactions between these can help inform PES mechanisms in relatively understudied ecosystems such as those in Palawan. Additionally, it is also important to identify how different factors (topographic, bioclimatic, and habitat structure) influence the species richness of threatened, range-restricted, and habitat-specific avian species.

This study investigates the relationship between AGB and avian biodiversity parameters in the Victoria-Anepahan mountain range in Palawan, the Philippines. Palawan, unlike other islands in the Philippines, is biogeographically part of the Sundaland. In spite of the Sundaic nature of the species composition, there is a considerable overlap between the Sundaland and the Philippines biota, which contributes to the unusual species makeup and endemism in Palawan [31]. Also, several International Union for Conservation of Nature (IUCN)-listed and endemic bird species are found in the study area [32]. Endemic [33] and rare/threatened [34,35] avian species are especially vulnerable to disturbances. Hence, it is imperative that the role of different factors, both natural and anthropogenic, in influencing the species richness of both endemic and IUCN red listed birds be quantified in order to inform conservation management.

In this study we (a) examined the degree of overlap between avian species richness (of both IUCN red listed and endemic birds) and AGB at 1-km resolution; (b) identified factors influencing local species richness of IUCN red listed and endemic avian species at 1-km resolution; and (c) indicated the extent to which variation in AGB influences avian species richness for both IUCN red listed and endemic birds. This research has also been used to build a local-scale AGB map using locally collected field data in conjunction with optical and ALOS-PALSAR data. This in turn allowed us to evaluate the efficacy of remote sensing data to predict the AGB of a closed canopy lowland forest ecosystem and to help calibrate a previously developed global scale 1-km pan-tropical AGB map [17].

## Methods

### Study area

**Fig 1:**
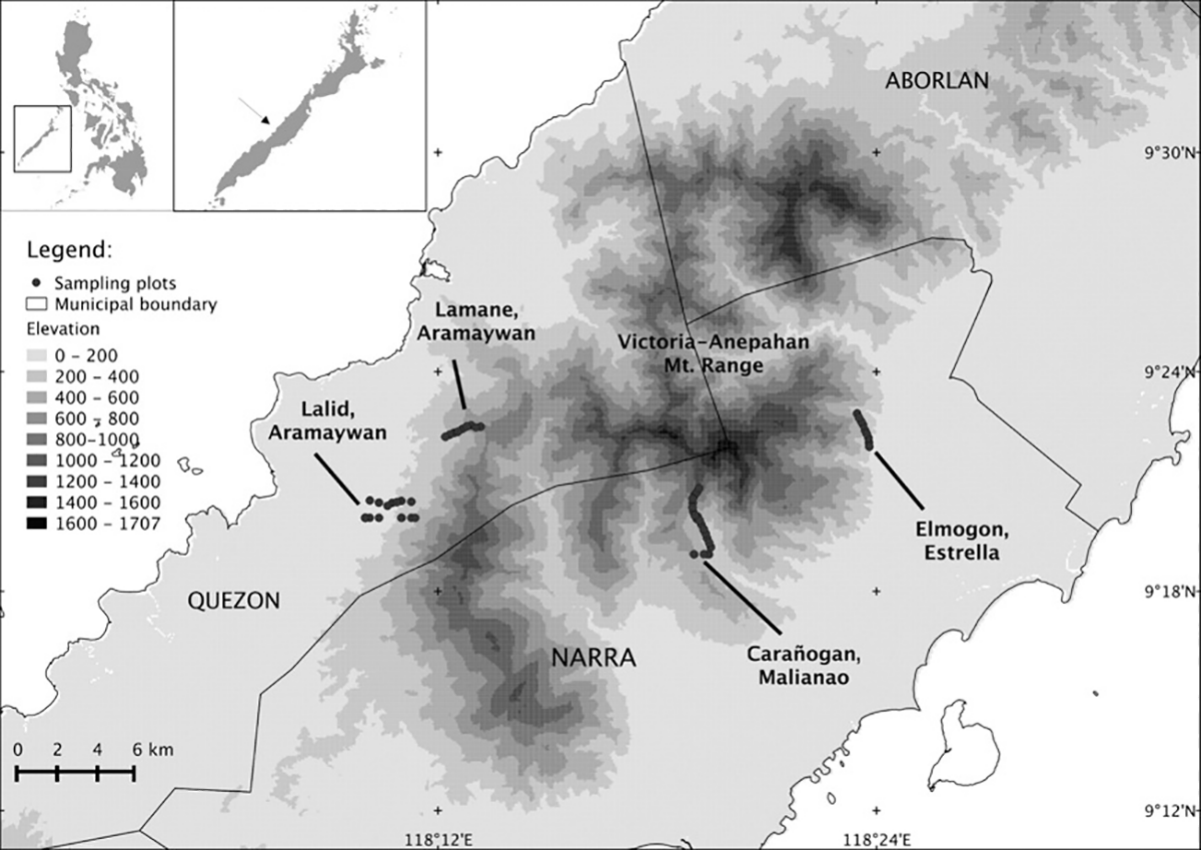
Location of the study area in the Victoria- Anepahan ranges, Palawan (*ASTER GDEM is a product of NASA and METI*) [39].

This study covers 951.67 km^2^ located within the Victoria-Anepahan mountain range, Palawan Island in the Philippines (Fig 1). The study area is a REDD+ pilot project site implemented by a partnership of non-government organizations and government agencies and was selected as a sub-national unit for testing the implementation of REDD+ in the Philippines [36]. Additionally, owing to the presence of many endemic avian species, the entire Palawan Island has been designated an Endemic Bird Area (EBA) and the Victoria-Anepahan range is designated a Key Biodiversity Area [37] and an Important Bird Area [38].

Palawan is regarded as having the highest forest diversity in all of the Philippines [32], where the average annual rainfall varies from 1600 mm to 3000 m, while the average annual temperature ranges from 26 to 28 degrees Celsius [40]. While montane forests dominate higher elevations, virtually undisturbed lowland evergreen tropical forests can be found around the foothills [32]. The area’s unique geology has resulted in higher elevations running down the “backbone” of Palawan and elevations reaching up to 1700 m [31]. Palawan has been under a commercial logging ban in maximum protected forests since the implementation of the Philippine Republic Act 7611, also known as the Strategic Environmental Plan (SEP) for Palawan Act of 1992. This act is one of the reasons why numerous forests in the area are relatively undisturbed, although forest edges have been encroached upon over the past decade. Palawan is among the last forest frontiers of the Philippines: from 1990–2000, Palawan had a deforestation rate of 0.07% while the Victoria-Anepahan region had a deforestation rate of 0.04% [41]; from 2007–2010, total forest loss was estimated at 48.64 sq. km in Victoria-Anepahan range [40]. Despite the logging ban, the Victoria-Anepahan range is currently under threat from extractive industries such as mining and illegal harvesting of forest products [42]. Oil palm also threatens forests and the traditional livelihoods of the indigenous communities in the area. Reducing contemporary deforestation rates by even 5–15% in areas of the Philippines such as Palawan through REDD+ could save substantial carbon emissions and provide a significant source of funding for local conservation [36]. Hence, even though the current rate of forest loss is low, it is imperative that PES mechanisms such as REDD+ and payments for biodiversity conservation be implemented in order to preserve the unique flora and fauna of the region into the future.

### Collection of field data

Field data collection was conducted from June to August 2013 by Fauna & Flora International (FFI)–Philippines Programme under the PSCD SEP Clearance REDD-022213-001 and Wildlife Gratuitous Permit GP 2013–02. Transects were placed using random stratified sampling to sample representative areas of the specific habitat type(s) found in the study area (primary old growth forests and recently logged secondary forest) [40].

Forest mensuration data were collected over four transects, covering a distance of two kilometers each. Forest inventory plots were established at 250 m intervals along each transect. Each 2-km transect comprised of nine 0.25 ha square plots (50 m^2^). Two transects were located in primary forests and two in secondary forests. In all plots, tree diameter, canopy cover and dead wood were recorded [40]. Using diameter tapes, trees ≥ 30 cm diameter at breast height (DBH) were measured within the 0.25 ha plots. These DBH data were used to derive AGB estimates at the hectare scale using the DBH-only allometric equation [43].

Canopy cover estimates were derived using a vertical densitometer at 0.25 ha scale [40]. To measure canopy cover, a diamond plot configuration was established within each 0.25 ha square plot, wherein the corners of the diamond plot bisected the sides of the square plot [40].

### Remote sensing (RS) data

Raw Landsat data at 30 m resolution from August 2014 was converted to reflectance values using ClasLite [44]. ClasLite applies sensor offset/gain to covert the raw Digital Number (DN) values to radiance values. Surface radiance values are converted to top-of-the-atmosphere reflectance values by passing through a 6S atmospheric correction model [44].

ALOS-PALSAR L-band data (from 2015) were acquired through the Japan Aerospace Exploration Agency [45]. ALOS-PALSAR data (Grid 28, N 10–15 and E 115) have a spatial resolution of 25 m, and the L-band has a wavelength of 24 cm allowing it to capture canopy structural information. Dual band polarization was used by the sensor in order to obtain the data in both HH (horizontal transmit-horizontal receive) and HV (horizontal-vertical) polarization modes. HH data are sensitive to horizontal volume scattering by canopy while HV data are sensitive to variation in vertical forest structure components such as height [46]. HH and HV data are complementary in mapping forest structure patterns. Variation in canopy structure and volume (arising out of factors such as forest degradation) are reflected in the backscatter values of these data, making them effective for quantifying forest degradation and separating degraded forests [47]. Inclusion of both HH and HV data significantly helped improve the accuracy of AGB models for a mixed forest ecosystem in Indonesia [48]. Filtering was implemented using the ENVI software with the view of reducing the speckles while preserving image texture. SAR DN data was converted to normalized radar cross section using the following equation [49]:

$$\sigma^{0}=10∙\log_{10}\left(DN^{2}\right)+CF$$ (1)

Here, *σ*^*0*^ is the normalized radar cross section (backscatter coefficient) and the value of CF (calibration factor) is– 83. The correction was implemented on both HH and HV data. HV is a much stronger predictor of AGB variation than HH-derived metrics [50,51,49]. The Radar forest degradation index (RFDI) is a quantitative measure of forest degradation and was computed by the formula (HH-HV)/(HH+HV) [50].

### Deriving texture measures from RS data

Image texture relates to the structural and spatial nature of the objects/scene being captured and it is composed of specific regions of well-defined characteristics such brightness, color, shape [52]. GLCM is a widely used method for quantifying image texture, assuming that texture is an innate property of all surfaces which can be captured by remote sensing imagery [53]. Texture contains information about the structural arrangement of the target surfaces/objects and their relationship with the surrounding environment. The practical implementation of GLCM statistically describes the variation in pixel values and/or relationship between the pixels. Specifically, this algorithm seeks to capture the tonal variability of pixels by calculating both first order and second order statistics [19]. First order statistics quantify the tonal variation of a given pixel (while ignoring its relationship with its adjacent pixels). Second order descriptive statistics are based on relationships among pixels, i.e., frequency of associations between the brightness of adjacent pixels [19,22]. GLCM texture variables (S1 Table) were extracted for both Landsat reflectance data and ALOS-PALSAR HH-HV data bands.

### Statistical modelling of AGB

Texture variables (derived from Landsat and ALOS PALSAR HV polarization), landscape scale canopy height (taken from Simard et al., [54]), topographic slope and ground-measured canopy cover were used as predictor variables for modelling AGB variation across the study area by implementing a log-log linear regression approach and a Random Forests (RF) machine learning approach.

Previous research has described the relationship between field measured AGB and predictor variables using a power law form [44]. The power law form is expressed in terms of a log-log linear relationship:

ln(AGB)=a+b×ln(PredictorVariable)+e (2)

This equation has been used in other studies to associate field AGB with both LiDAR-derived canopy heights [55] and aerial imagery-derived canopy cover [56]. We have implemented log-log linear regression using height and canopy cover individually and with each other similar to Réjou-Méchain, et al. [55] and Singh et al. [56].

The RF algorithm was used to select the most important variables from the texture variables, height and canopy cover with the view of building a predictive AGB model. The RF algorithm carries out recursive data partitioning to build many tree-based decision models, of which are brought together as an ensemble to carry out predictive modelling [57]. RF modelling works well with correlated predictor variables and makes no assumptions about underlying data distribution. Ten-fold cross validation was implemented to prevent over-fitting and to select the most appropriate model when using small sample sizes [58]. In 10-fold cross-validation, the original sample is randomly partitioned into 10 equally-sized subsamples; 9 subsamples are used as training data, and the remaining single subsample is validation data for testing the model. Each of the 10 subsamples is used once as the validation data, and the cross-validation process is repeated 10 times to reduce variability among the results. Results are then combined to produce a single error estimation. The advantage of 10-fold cross-validation is that all the samples in the dataset are eventually used for both training and testing [59].

Prior to modelling, highly correlated predictor variables (r≥0.75) were identified and removed [60] and were implemented using the caret package of the R programming language. For quantifying variable importance, error rate estimated from out-of-bag data were used to rank the predictor variables by their capacity to predict AGB. This out-of-bag error, also called out-of-bag estimate, is a method of measuring the prediction error of random forests [61]. The most parsimonious model was selected and used for building an AGB map at local scale. Predicted AGB values were compared with field AGB values using Pearson’s correlation, RMSE, MAE and %bias. The local scale AGB map that was built using field data and remote sensing texture variables was used to improve the efficacy of the 1-km Avitabile AGB mapped [17] by using the method of Langner et al. [62]. Correlation coefficients between the two AGB maps were calculated over kernel windows of varying size (4×4 pixels). For each kernel pixel center, the mean weighted values of different kernel sizes were selected to account for the existence of homogeneous areas at different scales. The derived weighted values provided the relative proportions of the sum of correlation coefficients for the 1-km AGB input map at pixel level.

### Identifying the factors influencing avian species richness

A combination of bio-climatic [17], topographic [28,63,25], habitat quality [50,64], and anthropogenic disturbance [65,66,67] related variables were used in addition to AGB to model species richness of IUCN conservation dependent and endemic avian species (Table 1).

**Table 1:** List of variables used as predictors.

Variable Name	Variable Description
**AGB**	Local scale AGB map combined with a 1 km resolution pan-tropical map to generate intermediate resolution AGB map, which captures AGB variation therein.
**Euclidean distance from Roads**	Road location data used to generate a raster for distance to the nearest road using ArcGIS 10.3.1. Distance from roads is known to influence avian communities in the Amazon.
**Euclidean distance from Rivers**	River location data were used to generate a raster for distance to the nearest road using ArcGIS 10.3.1.
**Elevation**	Represents surface terrain and expressed in meters above sea level
**Slope**	Derived from elevation data. Measures steepness and topographic variability. Known to be an important determinant of avian species richness.
**Aspect**	Derived from elevation data. Measures steepness, topographic and terrain variability of a given area. Known to influence species richness.
**RFDI**	Computed from the HH and HV polarizations. Is useful for distinguishing between the different vegetation types and levels of degradation.
**Landscape diversity index**	Quantifies diversity of different habitat/forest types in an area and is an important determinant of species richness. An important descriptor of landscape scale habitat condition. Land cover map of the study area at 300 m resolution was downloaded from ESA, and IDRISI software was used for deriving rasterized landscape diversity (S2 Fig).
**Landscape fragmentation index**	A measure of landscape spatial heterogeneity; higher values = greater habitat heterogeneity. Is an important descriptor of landscape scale habitat condition. Land cover map of the study area was downloaded from ESA, and IDRISI software was used for deriving rasterized habitat fragmentation (S2 Fig).

Variables were taken as predictors against the response variables of species richness of IUCN listed conservation-dependent and endemic bird species. Support vector regression (SVR) was used to identify the importance of individual predictor variables by projecting the input variables into a higher dimension in order to account for nonlinearity and complexity present within the ecological data. This allows the data space to function as a linear system while SVR focuses on finding a hyper-plane that may predict the distribution of information [68]. SVR models have better generalizability and lower risk of over-fitting [69], do not rely on statistical distributions of underlying data, and have no underlying requirement for independent data, allowing SVR to overcome auto-correlation [69]. In all, 1500 pixels were used for analysis.

Model fitting, testing and analysis of variable importance were performed using the ‘caret’ package of R [60]. A 10-fold cross-validation was employed for preventing over-fitting. In this, variable importance is tested by evaluating the relationship between each predictor variable and the outcome/response variable by fitting a loss smoother. The R^2^ is calculated for each of these models against the intercept-only null model, which is a relative measure of variable importance. The importance of variables in determining the variation in species richness was quantified at 1 km resolution. The direction of predictor value influence was quantified using correlation analysis. Further, univariate response curves of the six most influential predictor variables were derived using partial dependence plots, that help visualize non-linear and complex relationships, along with the direction of relationship between the response and the given predictor variable [70]. Relationships were quantified using the randomForest package to visualize the relationship between the response and the predictor variables and how the response variable changes with the change in the predictor variable [71].

### Species richness mapping

The ‘letsR’ package in the R programming language [72] was used to generate species presence-absence matrices from the geographical distributions of birds that coincide within the study area. Geographical distributions of birds were extracted from the Birdlife database and NatureServe database [73]. This included all species listed in the IUCN Red List and endemic birds (S2 Table). Presence-absence values for the study area were summed to obtain a species richness raster at 1 km resolution, and were further refined to obtain the species richness of Vulnerable (VU), Endangered (EN), Critically Endangered (CR), Near Threatened (NT), and endemic birds. In our study, we used bird species distributions, which are well-studied when compared to other taxonomic groups. We also restricted our dataset to include only endemic and threatened birds, which have smaller geographical ranges sizes (which make their cell size necessarily finer resolution).

### Spatial congruence

Correlations between AGB and all IUCN-listed bird species richness and AGB-endemic bird species richness were derived. The impact of spatial autocorrelation on effective degrees of freedom was accounted by implementing Dutilleul’s methodology [74]. Hotspot analysis was conducted by Getis-Ord Gi analysis in ArcGIS 10.3 [75], identifying statistically significant locations of “hotspots” where variables are spatially clustered. Z scores or p-values were also computed to indicate statistically significant spatial clusters of high and low variable values, i.e. “hotspots” and “coldspots” [76], with hotspots showing spatial clustering of high AGB values (>180 Mg ha^-1^) and high IUCN listed conservation dependent and endemic bird species richness [77]. Getis-Ord Gi calculates a standardized Z-score for each AGB and species richness value, which identifies the magnitude of deviation thus allowing us to determine the spatial clustering of small and large values [78]. Spatial congruence between the hotspots of species richness and AGB was computed by overlaying the species richness and AGB hotspot maps and computing the % area overlap.

## Results

### AGB mapping of the study area

The strength of association between predicted and actual AGB was 0.9, with RMSE being 31.05 Mg ha^-1^ and the mean absolute error being 24.02 Mg ha^-1^. Model %bias was 1.6, indicating a slight over-estimation of the predicted AGB values. Predicted AGB ranged from 32–238 Mg ha^-1^ (S1 Fig). On the other hand, the AGB values in Avitabile’s map ranged from 0 to 508 Mg ha^-1^ [17]. However, the fused map obtained after conducting pixel-based fusion between the two maps had AGB values ranging from 30–364 Mg ha^-1^, which captures the range of field AGB values better than the input maps individually. Landsat-based texture models of AGB had far lower predictive ability than this model and were thus not retained for further analysis (S3 Table). Additionally, models developed using LiDAR-derived canopy heights within the study area, field-measured canopy cover, and a combination of both had comparatively lower predictive ability and thus were not examined further (r = 0.78, 0.79, 0.89 respectively). Log-log linear regressions had the lowest predictive power. The canopy-only model had an adjusted R^2^ value of 0.34, the height only model had an adjusted R^2^ value of 0.46 and the model with both variables had an adjusted R^2^ model of 0.51. Hence, these models too were not explored further.

### Congruence between AGB and avian species richness

**Fig 2:**
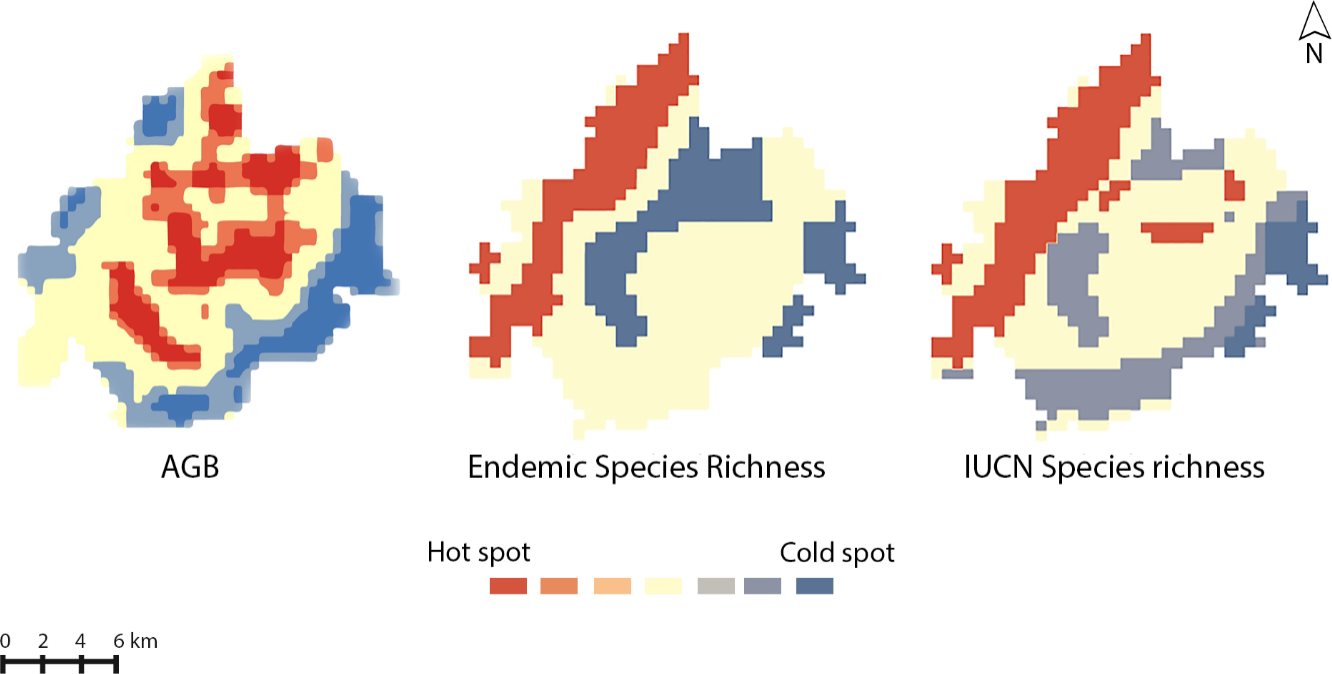
Areas of high and low AGB and avian species richness clustering.

A weak positive correlation existed between species richness of IUCN listed conservation dependent birds and AGB (r = 0.23). Species richness of endemic birds and AGB showed a weak negative correlation (r = -0.17). The p value was greater than 0.05 for all the cases indicating the possible non-significance of these associations. In addition to correlation analysis, hotspot analysis was conducted to identify the spatial clustering of species richness and AGB (Fig 2).

Overlay analysis showed that there was a 13% overlap between areas of high AGB values and high IUCN listed conservation dependent bird species richness. The spatial overlap between areas of high AGB and high endemic bird species richness was 8%. Overlay analysis showed that there was a 36% overlap between areas of low AGB values and low IUCN listed conservation dependent bird species richness. The spatial overlap between areas of low AGB and low endemic bird species richness was 12%.

### Factors influencing avian species richness

The variable importance for explaining the variation in the IUCN listed conservation dependent avian species richness estimated by the SVR model is presented in Table 2.

**Table 2:** Importance of predictor variables for explaining the variation in IUCN listed conservation dependent bird species richness.

Variable	Variable Importance at 1-km
**AGB**	100.0
**Aspect**	90.3
**Euclidian distance from road**	87.6
**Altitude**	58.6
**Slope**	57.4
**RFDI**	56.2
**Landscape diversity**	20.4
**Landscape scale fragmentation index**	20.4
**Annual rainfall (mm)**	15.9
**Euclidian distance from river**	7.2
**Mean temperature**	5.1
**Precipitation Seasonality**	0.0

**Fig 3:**
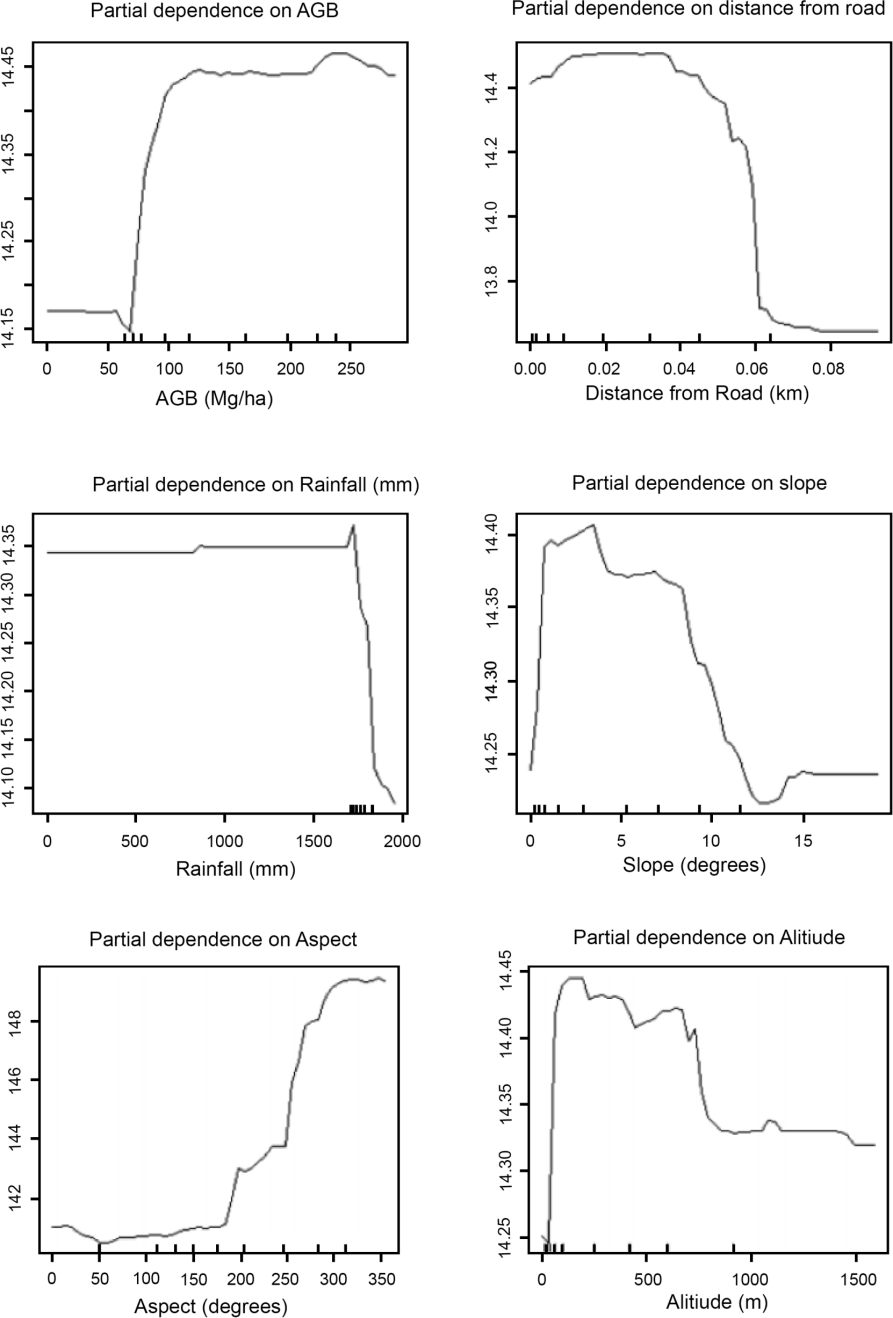
Partial dependence plots at 1-km scale for IUCN species.

AGB was the most important variable for explaining variation in IUCN red listed avian species richness, followed by aspect and the Euclidian distance from roads. Correlation analysis also showed that IUCN red listed avian species richness was positively associated with AGB, (r = 0.52, p<0.01), and is further confirmed by the univariate response curves developed for this. Another important variable in this model is aspect. The curve for aspect indicated that the number of IUCN birds stagnates from zero aspect to about 160 and began to rise sharply up to an aspect value of ~290. The number of IUCN birds began to rise from areas of AGB value of about 70 Mg ha^-1^ and increased steadily up to about value 82 Mg/ha the gradually up to about 245 Mg ha^-1^. The third most important variable is distance from roads. The values of IUCN bird species richness increased steadily with increasing distance and then leveled off. From these it may be inferred that at a 1-km scale, IUCN red listed avian species richness was best supported in areas with higher AGB values and greater distance from roads (Fig 3).

**Fig 4:**
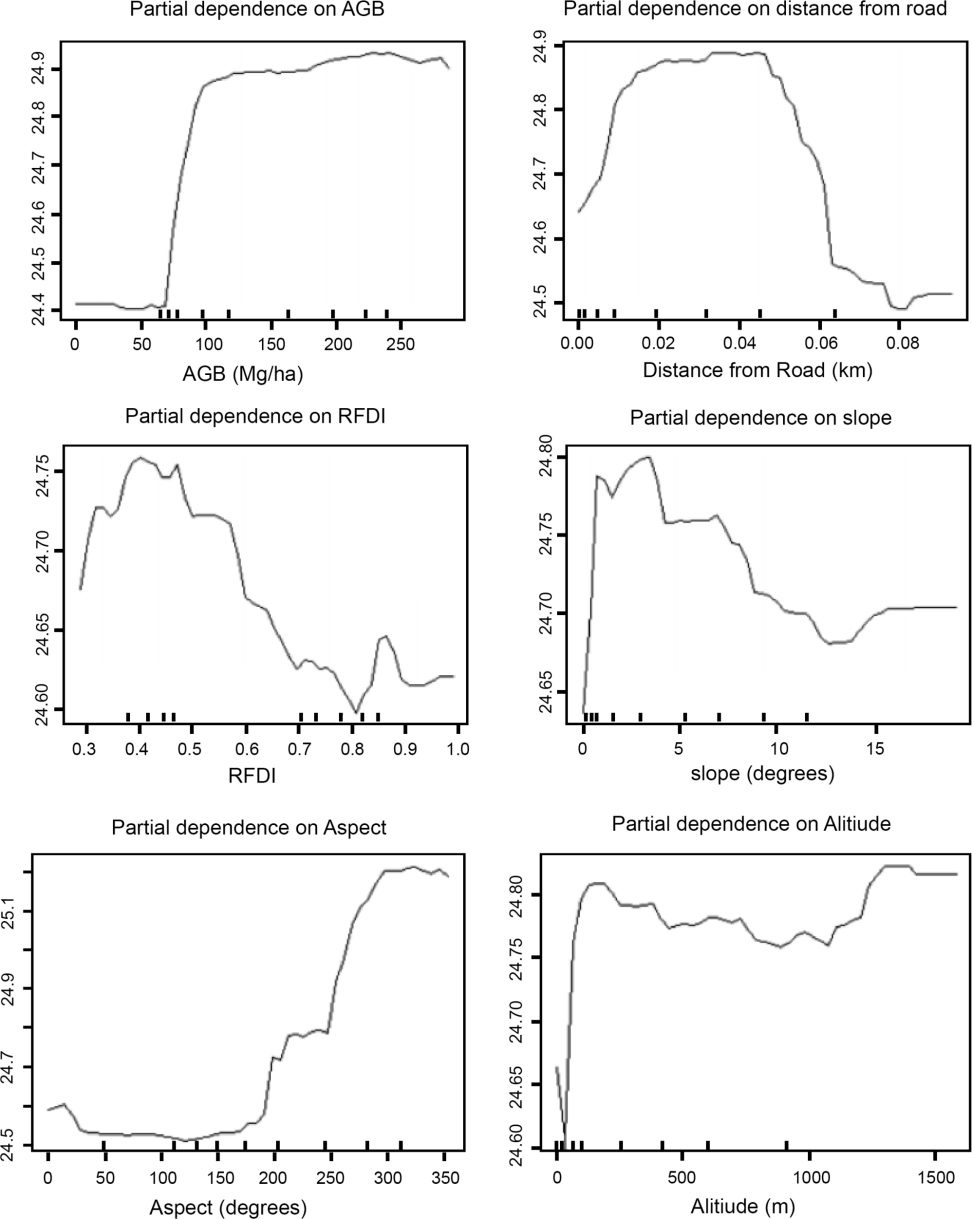
Partial dependence plots at 1-km scale for endemic species.

Distance from roads was the most important variable for explaining variation in endemic species richness values. The importance of AGB in explaining the variation in species richness was 62.72%. However, topographic variables such as aspect, altitude, and slope had a greater explanatory power than AGB. While slope had a negative association with endemic avian species richness, aspect had a positive association with endemic avian species richness. AGB, on the other hand, was weakly associated with endemic species richness. The partial dependence plots indicate that endemic bird species richness began to increase gradually from aspect value 180 to about aspect value of 270 (Fig 4). There was a negative relationship between endemic species richness and distance from roads, indicating the farther the distance from roads, the greater the species richness. RFDI was negatively associated with endemic avian species richness indicating that higher levels of forest degradation were inversely related to the species richness.

The relative variable importance for explaining the variation in the endemic avian species richness estimated by the SVR model is shown in Table 3.

**Table 3:** Relative importance of predictor variables for explaining the variation in endemic bird species richness.

Variable	Variable Importance
**AGB**	62.7
**Aspect**	78.7
**Euclidean distance from road**	100.0
**Altitude**	65.3
**Slope**	27.9
**RFDI**	6.7
**Landscape diversity**	0.5
**Landscape scale fragmentation index**	0.00
**Annual rainfall (mm)**	45.0
**Euclidean distance from river**	16.7
**Mean temperature**	6.7
**Precipitation Seasonal**	0.4

## Discussion

### AGB mapping

The most parsimonious predictive model of AGB comprised of second moment texture band of HH, correlation texture band of HH and homogeneity texture band of HV, similar to previous studies [22] where the inclusion of HH and HV variables improved AGB estimates. Models comprising of Landsat texture based predictors and forest biophysical parameters (canopy cover and height) did not perform as well as the selected ALOS-PALSAR texture based AGB model. This may be attributed to the inherent nature of optical and radar data, particularly their reduced ability to predict values as AGB increases. It has been suggested L-band SAR data start losing their sensitivity to AGB beyond 100 t ha^-1^ [50] as denser canopies lead to signal attenuation and associated loss of sensitivity [79]. This is consistent with the existing literature, where machine learning mangrove AGB models that used optical data only underestimated AGB at higher levels [77]. However, the underlying rationale for generating AGB models was to demonstrate how machine learning may be employed for producing predictive AGB models using a combination of field and satellite data. Efficacy of machine learning models such as RF and SVR, among others, for predicting AGB was examined. It was discovered that machine learning models produce robust AGB estimates, especially for ecosystems where canopy cover values vary from 40%-60% [80]. A comparison of the efficacy of ML approaches for estimating AGB in degraded tropical peat forests showed that machine learning approaches produce more robust AGB models than linear regression [81]. This is the case in this study as well, where machine learning models performed better than the traditional log-log linear approach followed by previous studies. This may be because machine learning models account for inherent data complexity and are unaffected by the underlying data distribution [44,82,55].

GLCM texture variables derived from SAR and Landsat data could also successfully produce AGB estimates, as per other studies where artificial neural networks produced robust AGB estimates for similar tropical forest ecosystems in Malaysia, Thailand and Brazil [21]. SAR backscatter data alone had a weak strength of association with field AGB. GLCM texture measures have been derived from remote sensing data of varying spatial resolutions (from 30 m Landsat to 50 cm Digital Globe data) to predict AGB variation in different tropical ecosystems with varying levels of accuracy. GLCM measures derived from high resolution SPOT-5 image obtained over a tropical forest ecosystem in Chiapas (Mexico) had a moderate strength of association with field AGB [83], though, GLCM texture derived AGB from Malaysian Borneo (for forests that had undergone varying levels of logging) had a very strong strength of association with field AGB values [19]. However, it must be noted that the latter resulted with a more reduced canopy cover as compared to our study area. Inclusion of canopy cover with texture measures significantly improved the predictive ability of AGB models in an open canopy ecosystem in Cambodia. Log-log regression model containing canopy as the only predictive variable also produced robust AGB estimates for this ecosystem [56]. This may be explained on the basis of the fact that field measured AGB and ground canopy cover in the Cambodian woodlands had a strong strength of association (r = 0.69) [56]. For our study area, ground measured canopy cover had a relatively low strength of association with field measured AGB (r = 0.33, p<0.01). This also explains why models containing canopy cover (both log-log and RF) had a comparatively lower predictive ability. However, GLCM texture derived variables helped produce relatively robust AGB models. On the basis of this, it may be inferred that GLCM derived texture measures can help produce robust AGB estimates across a variety of tropical ecosystems ranging from open canopy woodlands to closed canopy, virtually undisturbed forests such as in Victoria-Anepahan range. Our research also indicates that machine learning models can produce robust AGB estimates for undisturbed ecosystems with high canopy cover as well.

One of the major drawbacks of our local scale AGB model is biomass saturation that occurs with optical and radar data at higher values. Further, GLCM texture measures are more accurate in predicting AGB for degraded forest systems as opposed to intact ones [20]. While Cutler et al. [21] did not explicitly discuss the problem of biomass saturation in their research, our findings are similar to studies where ALOS-PALSAR-based estimates experience saturation at similar AGB values such as in the tropical forests of eastern India [84]. Further, the AGB map developed by Avitabile et al. [17] had the maximum predicted AGB value of around 500 Mg ha^-1^, which is much higher than the maximum AGB value recorded in this study. This may be owing to the fact that no field data from Palawan were used for calibrating the 1 km resolution pan-tropical map [17]. However, the fused map produced by combining both local and global scale AGB maps captured the variation in AGB values of our study area better than the input maps and captures the spatial distribution of the bespoke AGB values. On further examination, it was discovered that 92% of AGB values greater than 200 Mg ha^-1^ fall in forested areas (located in the interior of the study area), with areas > = 180 Mg ha^-1^ generally regarded as high AGB areas in the Asian tropics [85].

### Congruence between areas of high AGB and high avian species richness

Correlations suggest a limited strength of association between AGB and species richness of endemic and IUCN listed conservation dependent species richness. This may be attributed to the inability of correlation analysis to capture complex processes at a large scale. These findings are similar to a recent national level study conducted in Indonesia, which indicated a weak negative correlation between AGB stocks and species richness [8].

An examination of spatial congruence between areas of high AGB and high avian species richness revealed a spatial overlap between the areas of high AGB and high IUCN listed conservation dependent species richness (13%) and areas of high AGB and endemic avian species richness (8%). However, there was a greater magnitude of spatial overlap between the areas of low AGB and low IUCN listed conservation dependent species richness (36%) and areas of low AGB and endemic avian species richness (12%), suggesting that areas with lower AGB values correspond closely with areas of low avian species richness. The study area has a high proportion of undisturbed forests that are reservoirs of both high biodiversity and high AGB values [86,87]. Research by Mallari et al. [88] indicates that even mild forest degradation has detrimental effects on the persistence of Palawan’s endangered and endemic avian species richness. Low AGB values are indicative of forest loss and severe degradation [89,82] and this in turn may be associated with lower avian species richness owing to a loss or degradation of important habitats that bird species rely on.

Our study does not make claims of causality between AGB and species richness. It is possible that as we scale up to the national scale and include more disturbed forests we may find a decline in spatial congruence between species richness-based biodiversity values and AGB, in line with the findings of Murray et al. [8]. Further, high AGB forest ecosystems (such as rapidly growing plantations) could have lower species richness [90]. Hence future research should examine relationships between AGB and species richness, while accounting for factors such as forest degradation.

The criteria of designating an area as one of high biodiversity value are still ambiguous. At a continent-wide scale, an overlap of four global biodiversity priority schemes is used as a criterion for designating an area as one of high biodiversity value [85]. However, global biodiversity priority schemes do not shed much light on biodiversity values at regional or landscape scales, or provide guidance on where cut-offs are most appropriate for designating an area of high species richness or high biodiversity value. While the best way of categorizing the biodiversity value of an ecosystem remains highly subjective [91] and may vary at different spatial scales, we have presented a way of using the existing databases for quantifying species richness in a data-sparse species rich tropical forest. Additionally, we have sought to identify areas where there is a concentration of higher AGB values and high species richness via the Getis-Ord hot-spot analysis. This approach has not been widely used in conservation studies. It was previously deployed for identifying statistically significant richness clusters of threatened and rare orchids in the Neotropics [75]. By implementing this approach, we spatially located areas of high AGB storage and species richness and areas where they overlap. Such a mapping endeavor has the potential to inform practical conservation on the ground and is more informative than a correlation-only based approach. This examination certainly sheds more light on the spatial patterns of AGB and species richness distribution than ordinary correlation analysis carried out both in this research and the work by Murray et al. [8]. However, correlation analysis is not sufficient to capture the complex interplays and associations that may exist between AGB values and avian species richness. These techniques must be used in conjunction with ground data to refine richness estimates and inform conservation priorities in species-rich forests such as those in Palawan.

Moderate links between AGB and avian diversity (as opposed to an expected strong relationship) has important implications for conservation planning. Earlier works have discovered that the target areas for REDD+ payments in Indonesia contained globally threatened mammal species (such as the Bornean orangutan, Borneo pygmy elephant) in Kalimantan, so that carbon payments could facilitate the conservation of these species [92]. These conflicting results indicate that spatial congruence between areas of high AGB and high species richness is not consistent across different tropical forest ecosystems and these patterns need to be examined in multiple areas [93]. Weaker congruence between AGB and avian diversity at larger spatial scales, as shown in this study, suggests that challenges exist to the large-scale planning of carbon PES if biodiversity co-benefits are also a project objective [6].

### Factors influencing avian species richness

Species richness of both IUCN-listed conservation-dependent and endemic avian species in southern Palawan is influenced by a combination of topographic, bioclimatic, and habitat quality related variables. AGB and factors such as sources of anthropogenic disturbance also affect species richness. At the 1 km scale used here, AGB values are closely associated with variation in avian species richness of both IUCN-listed and endemic avian species richness. An examination of how avian species richness varies with AGB was conducted by Lees et al. [10] using linear regression in a mixed forest ecosystem in the Amazon rainforest, showing that a linear regression model with AGB as the only explanatory variable explained 70% of the variation in avian species richness and had a moderately high variable importance as compared to other explanatory variables [10].

Slope and aspect were significant predictors of endemic and IUCN species richness. The role of elevation gradients and its influence on species richness and community dynamics is well chronicled in literature [94]. Topography is a well-known driver of species richness in the tropical forests [95,96]. A previous study also discovered that topographic variables influence avian species richness at an intermediate scale of 1 km^2^ [27]. Neotropical research revealed that avian richness is extremely sensitive to variations in elevation and topography [97]. Aspect-related variables are also known to influence avian species richness, and were important drivers of avian species richness in this study. It was discovered that species richness on the external slopes of the Colombian Andes decreased with increasing elevation while richness and elevation had a hump-backed relationship on the internal slopes [98]. A similar situation of endemic species richness being affected by rising altitudes has been suggested by our research as well. Steeper slopes, especially in relatively low altitude tropical and sub-tropical ecosystems are known to harbor higher avian species richness [99]. It has been suggested that topographic heterogeneity and variations on altitudinal and slope patterns provide a niche to species and supports speciation [98].

Additionally, for both endemic and IUCN listed conservation dependent avian species richness, distance from roads was among the most important variables influencing the variation in species richness. This is consistent with previous research [100] which summarized the impacts of roads and linear clearings on tropical forests and other studies which indicate that road distance is important for explaining the variation in avian species richness and often has a negative impact on species richness and persistence [101,28]. Proximity to roads is a powerful landscape scale driver of forest loss [102] and is associated with declines in forest cover [103]. Road density increases over a 68 year period contributed to increasing forest fragmentation in Jamaica [104].

Recent research indicates that anthropogenic disturbances can accelerate avian species extinction, especially for rarer birds [34]. The vulnerability of birds to forest degradation is also reflected in the fact that higher RFDI values were associated with decreasing species richness at the 1 km scale. RFDI was previously used for monitoring and mapping forest degradation in tropical Africa [18]. This research establishes the potential of this metric for explaining the variation in avian species richness in a tropical forest.

### The role of spatial resolution

In this study, factors influencing avian species richness varied according to spatial scale. While habitat-related factors were important influencers at smaller spatial scales (less than 6 km resolution), factors relating to available energy drive avian species richness at larger spatial scales [24]. The question of spatial scale and resolution is an important consideration for studies like ours. Many factors influence the decision on the grid cell size and it is difficult to use a general number. The scale of species richness computation is very important and 100 km has been a widely used spatial resolution scale, though may be too coarse for capturing species richness in areas of high endemism and topographic patterns (such as mountains) that influence local species persistence [105]. Further, the 100 km scale was found to degrade the raw data and obscure patterns of local richness and diversity. It has been further suggested that local scale species richness mapping be undertaken at spatial resolutions <10 km [106].

At a 1 km scale, avian species richness was positively associated with AGB and negatively associated with forest degradation and disturbance. Endemic and threatened birds are highly sensitive to the slightest disturbance and prefer virtually intact forests [35]. Primary and/or virtually undisturbed forests of the Asian tropics have higher AGB values compared to disturbed forests [89]. Hence, high AGB values may be taken as a proxy for forest intactness. Thus, this study suggests that intact forests are important reservoirs of AGB storage and also provide vital habitat to endangered and endemic avian species.

## Conclusions

This research examined AGB, biodiversity distribution, and congruence across a landscape in Palawan using freely available remote sensing data. Combining AGB data from two different sources, including a combination of local-scale field data and texture metrics helped robustly capture AGB variation. Improving global scale AGB maps with local estimates can help inform the AGB storage values of different forest ecosystems.

The research has laid the first step in the spatial mapping of AGB and biodiversity (in this case, represented by avian species richness) of a vital and increasingly threatened ecosystem. Also, the research exhibited how available data repositories such as the BirdLife databases (among others) are important repositories of information that can shed light on species richness in data sparse areas. Although data mining of archival resources has not been considered extensively in ecological studies, this study presents a roadmap of using them in conservation management. Robust spatial congruence has been discovered between the areas of low AGB and low species richness. Moreover, AGB values play an important role in explaining the variation in species richness at 1-km for our study area. Thus, remaining forested tracts within the study area are an important reservoir for both carbon stocks and avian species richness. Further, both IUCN red-listed and endemic species are negatively influenced by anthropogenic disturbances, notably distance from roads. Hence, the long-term persistence of these species may require a strict protection of their habitat to reduce the impact of anthropogenic disturbances. While this study examines the factors influencing avian species richness in a relatively undisturbed ecosystem, it is also important to quantify habitat preferences of avian species in human modified tropical ecosystems that dominate much of tropical Asia. Conservation prioritization requires us to identify local and landscape variables that influence avian species persistence across ecosystems.

## Supporting information

S1 Fig Field AGB and predicted AGB. (TIF)

S2 Fig 1-km LULC map of the study area. Created using QGIS (QGIS Development Team, 2016.
(TIF)

S1 Table Texture variables derived from GLCM. (PDF)

S2 Table Classification of bird species. (PDF)

S3 Table Performance of landsat texture derived AGB models. (PDF)
