# Supplementary figures and images for: Spatial relationships between above-ground biomass and bird species biodiversity in Palawan, Philippines
Source: PLoS One. 2017 Dec 4;12(12):e0186742. doi: 10.1371/journal.pone.0186742 (PMC5714345; doi:10.1371/journal.pone.0186742)

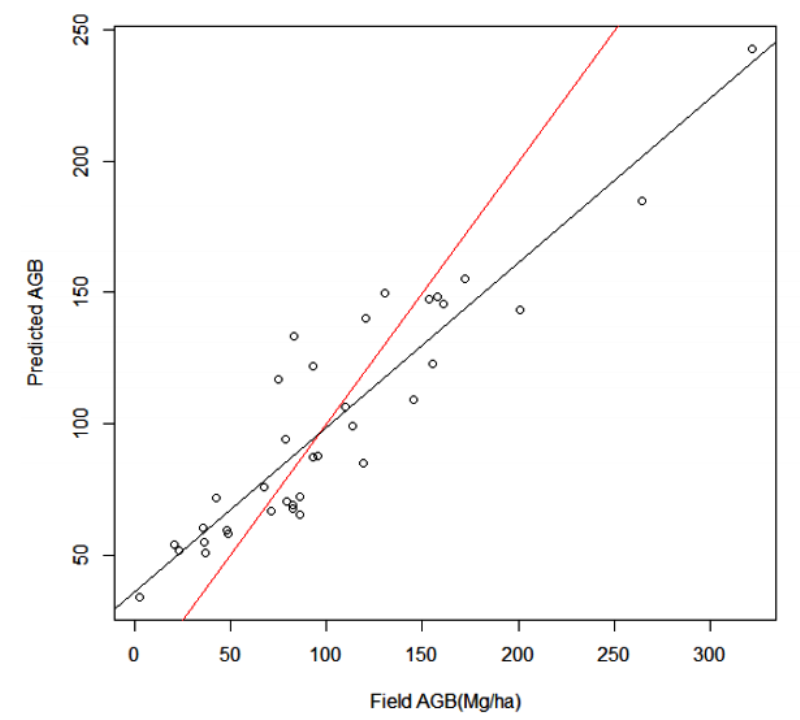

Supplement: S1 Fig — (TIF) [file pone.0186742.s001.tif]

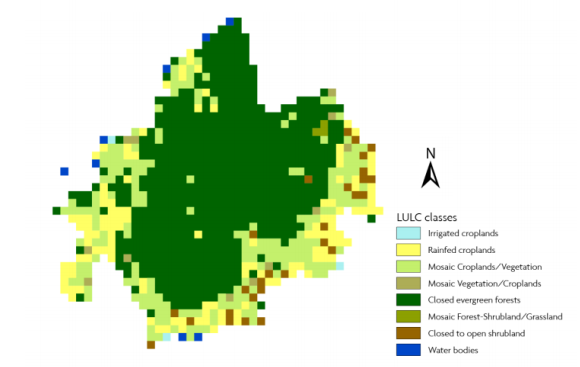

Supplement: S2 Fig — Created using QGIS (QGIS Development Team, 2016. (TIF) [file pone.0186742.s002.tif]
