# Supplementary material for: Spatial relationships between above-ground biomass and bird species biodiversity in Palawan, Philippines
Source: PLoS One. 2017 Dec 4;12(12):e0186742. doi: 10.1371/journal.pone.0186742 (PMC5714345; doi:10.1371/journal.pone.0186742)
Supplement: S1 Table — (PDF) [file pone.0186742.s003.pdf]

Supporting Information for

Spatial relationships between above-ground biomass and bird species biodiversity in Palawan, Philippines

S3 Table: Texture variables derived from GLCM (Singh et al., 2014; Gallardo-Cruz et al., 2012)

Texture Variable	Statistics	Formula	Description
Mean	First-Order	$MEAN = \sum_{ij=0}^{N-1} iP_{ij}$	Mean of the probability values from the GLCM. It is directly related to the image spectral heterogeneity.
Variance	First-Order	$VAR = \sum_{ij=0}^{N-1} P_{ij}(i - MEAN)^2$	Measure of the global variation in the image. Large values denote high levels of spectral heterogeneity.
Correlation	Second-Order	$COR = \sum_{ij=0}^{N-1} P_{ij}\left[\frac{ij - MEAN_{ij} - MEAN}{VAR}\right]$	Measure of the linear dependency between neighbouring pixels.
Contrast	Second-Order	$CONT = \sum_{ij=0}^{N-1} P_{ij}(i - j)^2$	Quadratic measure of the local variation in the image. High values indicate large differences between neighbouring pixels.
Dissimilarity	Second-Order	$DISS = \sum_{ij=0}^{N-1} P_{ij}(i - j)$	Linear measure of the local variation in the image.
Homogeneity	Second-Order	$HOM = \sum_{ij=0}^{N-1} P \frac{P_{ij}}{1 + (i - j)^2}$	Measure of the uniformity of tones in the image. A concentration of high values along the GLCM diagonal denotes to a high homogeneity.
Angular second moment	Second-Order	$ASM = \sum_{ij=0}^{N-1} {P_{ij}}^2$	Measure of the order in the image. It is related to the energy required for arranging the elements in the system.
Entropy	First-Order	$ENT = \sum_{ij=0}^{N-1} P_{ij} ln P_{ij}$	Measure of the disorder in the image. It is inversely related to ASM.

Singh, M., Malhi, Y. & Bhagwat, S., Evaluating land use and aboveground biomass dynamics in an oil palm–dominated landscape in Borneo using optical remote sensing. Journal of Applied Remote Sensing 8 (1), 083695:1-14 (2014).

Gallardo-Cruz, J.A., Meave, J.A., González, E.J., Lebrija-Trejos, E.E., Romero-Romero, M.A., Pérez-García, E.A., Gallardo-Cruz, R., Hernández-Stefanoni, J.L. and Martorell, C., 2012. Predicting tropical dry forest successional attributes from space: is the key hidden in image texture?. PLoS One, 7(2), p.e30506.
