# Supplementary material for: Spatial relationships between above-ground biomass and bird species biodiversity in Palawan, Philippines
Source: PLoS One. 2017 Dec 4;12(12):e0186742. doi: 10.1371/journal.pone.0186742 (PMC5714345; doi:10.1371/journal.pone.0186742)
Supplement: S2 Table — (PDF) [file pone.0186742.s004.pdf]

Supporting Information for

Spatial relationships between above-ground biomass and bird species biodiversity in Palawan, Philippines

S1 Table: Classification of Bird Species

Category	Species
**Vulnerable**	*Anas luzonica, Anthracoceros marchei, Egretta eulophotes, Ficedula platenae, Mulleripicus pulverulentus, Polyplectron napoleonis, Prioniturus platenae, Ptilocichla falcata, Streptopelia dusumieri*
**Near Threatened**	*Anhinga melanogaster, Anthreptes rhodolaemus, Calidris ruficollis, Caloenas nicobarica, Calonectris leucomelas, Charadrius peronii, Cyornis lemprieri, Dinopium everetti, Esacus magnirostris, Malacopteron palawanense, Oriolus xanthonotus, Otus fuliginosus, Otus mantananensis, Parus amabilis, Pericrocotus igneus, Stachyris hypogrammica, Tanygnathus lucionensis, Terpsiphone atrocaudata, Tringa brevipes*
**Critically Endangered**	*Cacatua haematuropygia, Fregata andrewsi*
**Endemic**	*Anas luzonica, Polyplectron napoleonis, Phapitreron leucotis, Ptilinopus occipitalis, Caprimulgus manillensis, Collocalia troglodytes, Aerodramus mearnsi, Hierococcyx pectoralis, Amaurornis olivacea, Nisaetus pinskeri, Otus mantananensis, Otus fuliginosus, Anthracoceros marchei, Cacatua haematuropygia, Prioniturus platenae, Rhipidura nigritorquis, Corvus philippinus, Terpsiphone cyanescens, Prionochilus plateni, Aethopyga bella, Chloropsis palawanensis, Pardaliparus amabilis, Orthotomus derbianus, Alophoixus frater, Iole palawanensis, Pycnonotus cinereifrons, Zosterornis hypogrammicus, Zosterops nigrorum, Malacopteron palawanense, Trichastoma cinereiceps, Ptilocichla falcata, Sarcops calvus, Kittacincla nigra, Cyornis lemprieri, Ficedula platenae*
