# Supplementary material for: Spatial relationships between above-ground biomass and bird species biodiversity in Palawan, Philippines
Source: PLoS One. 2017 Dec 4;12(12):e0186742. doi: 10.1371/journal.pone.0186742 (PMC5714345; doi:10.1371/journal.pone.0186742)
Supplement: S3 Table — (PDF) [file pone.0186742.s005.pdf]

Supporting Information for

Spatial relationships between above-ground biomass and bird species biodiversity in Palawan, Philippines

S2 Table: Performance of Landsat texture derived AGB models

Model	r	RMSE (Mg/ha)	MAE (Mg/ha)	%Bias
**Correlation of Band 3**	0.47	59.51	42.90	– 0.30
**Homogeneity of Band 4**	0.85	40.32	29.45	– 0.30
**Second moment of Band 4**	0.77	43.55	32.33	– 0.30
**Contrast of Band 5**	0.88	25.88	36.70	1.70
**All predictors combined**	0.90	35.86	27.13	– 1.00
